# Glycine receptor autoantibodies disrupt inhibitory neurotransmission

**DOI:** 10.1093/brain/awz297

**Published:** 2019-10-08

**Authors:** Sarah J Crisp, Christine L Dixon, Leslie Jacobson, Elodie Chabrol, Sarosh R Irani, M Isabel Leite, Guy Leschziner, Sean J Slaght, Angela Vincent, Dimitri M Kullmann

**Affiliations:** 1 UCL Institute of Neurology, University College London, London, UK; 2 Nuffield Department of Clinical Neurosciences, University of Oxford, Oxford, UK; 3 Department of Neurology, Guy’s and St Thomas’ NHS Foundation Trust, London, UK; 4 Department of Clinical Neuroscience, King’s College London, London, UK; 5 Wessex Neurological Centre, University Hospital Southampton NHS Foundation Trust, Southampton, UK

**Keywords:** glycine receptor, progressive encephalomyelitis with rigidity and myoclonus (PERM), stiff person syndrome (SPS), autoantibody

## Abstract

Chloride-permeable glycine receptors have an important role in fast inhibitory neurotransmission in the spinal cord and brainstem. Human immunoglobulin G (IgG) autoantibodies to glycine receptors are found in a substantial proportion of patients with progressive encephalomyelitis with rigidity and myoclonus, and less frequently in other variants of stiff person syndrome. Demonstrating a pathogenic role of glycine receptor autoantibodies would help justify the use of immunomodulatory therapies and provide insight into the mechanisms involved. Here, purified IgGs from four patients with progressive encephalomyelitis with rigidity and myoclonus or stiff person syndrome, and glycine receptor autoantibodies, were observed to disrupt profoundly glycinergic neurotransmission. In whole-cell patch clamp recordings from cultured rat spinal motor neurons, glycinergic synaptic currents were almost completely abolished following incubation in patient IgGs. Most human autoantibodies targeting other CNS neurotransmitter receptors, such as *N*-methyl-d-aspartate (NMDA) receptors, affect whole cell currents only after several hours incubation and this effect has been shown to be the result of antibody-mediated crosslinking and internalization of receptors. By contrast, we observed substantial reductions in glycinergic currents with all four patient IgG preparations with 15 min of exposure to patient IgGs. Moreover, monovalent Fab fragments generated from the purified IgG of three of four patients also profoundly reduced glycinergic currents compared with control Fab-IgG. We conclude that human glycine receptor autoantibodies disrupt glycinergic neurotransmission, and also suggest that the pathogenic mechanisms include direct antagonistic actions on glycine receptors.

## Teaser

Immunoglobulin G autoantibodies to glycine receptors are found in many patients with progressive encephalomyelitis with rigidity and myoclonus (PERM). Crisp *et al.* show that purified patient IgGs disrupt inhibitory neurotransmission in cultured motoneurons, and provide evidence for direct antagonistic actions on glycine receptors.

## Introduction

Over the past 15 years autoantibodies to extracellular epitopes of neuronal surface proteins have been described in patients with a spectrum of acquired neuropsychiatric diseases (Crisp *et al.*, 2016). The discovery of such autoantibodies has revolutionized neurological practice since, in contrast to autoantibodies against intracellular epitopes, these autoantibodies have the potential to be directly pathogenic and the associated diseases correspondingly often respond to immunomodulatory therapies. Knowledge that an autoantibody to a neuronal surface protein is pathogenic facilitates the prompt clinical recognition of patients likely to respond to early immunotherapy aimed at reducing the levels of the antibodies (e.g. plasmapheresis) and suppressing the pathogenic immune response. Nonetheless, responses to treatment may be incomplete and agents with substantial risk of adverse effects are often required, and in some cases prolonged periods of intensive care and rehabilitation are necessary before the treatment effects are seen. For individual patients, it is therefore crucial to establish a definite diagnosis of antibody-mediated neurological disease, ensuring that systemic and sustained immunotherapies are used if required. While specific autoantibodies to exposed epitopes in the CNS are identified in an increasing proportion of patients presenting with neurological disorders, it is sometimes unclear whether the autoantibodies are pathogenic or whether they occur as an epiphenomenon, for example as a result of neuronal damage due to degenerative processes or seizures (Crisp *et al.*, 2016; Dalmau *et al.*, 2017). While laboratory studies support a pathogenic role of autoantibodies to *N-*methyl-d-aspartate (NMDA) receptors (Hughes *et al.*, 2010; Planaguma *et al.*, 2015), α-amino-3-hydroxy-5-methyl-4-isoxazolepropionic acid (AMPA) receptors (Peng *et al.*, 2015; Haselmann *et al.*, 2018), gamma-aminobutyric acid (GABA) B receptors (Nibber *et al.*, 2017), and potassium-channel associated proteins such as leucine rich glioma inactivated 1 (LGI1) (Petit-Pedrol *et al.*, 2018), whether autoantibodies to glycine receptors interfere with normal neuronal signalling remains under-explored.

Glycine receptor autoantibodies were first identified in a patient with progressive encephalomyelitis with rigidity and myoclonus (PERM) (Hutchinson *et al.*, 2008). This disorder is characterized by a combination of muscle stiffness and spasms, myoclonic jerks, exaggerated startle responses (hyperekplexia), autonomic dysfunction, cognitive impairment and brainstem signs (such as oculomotor dysfunction and disturbance of respiratory rhythms) (Barker *et al.*, 1998; Meinck and Thompson, 2002; McKeon *et al.*, 2012). The symptoms of PERM could be explained by a failure of inhibitory neurotransmission in the brainstem and spinal cord. Indeed it was the clinical similarity between PERM and inherited deficiencies in glycinergic neurotransmission—the hereditary hyperekplexias (Shiang *et al.*, 1993; Langosch *et al.*, 1994; Bode and Lynch, 2014)—that led to the first identification of glycine receptor autoantibodies in PERM (Hutchinson *et al.*, 2008). This and 43 other patients were described, with a wider spectrum of clinical features including some with encephalopathies (Carvajal-Gonzalez *et al.*, 2014). Approximately half of patients with a clinical diagnosis of PERM had these antibodies (Martinez-Hernandez *et al.*, 2016) and they have also been described in a minority of patients with stiff person syndrome (SPS), who experience muscle stiffness and spasms but typically lack the brainstem signs that are characteristic of PERM. As more patients have been tested, serum and CSF glycine receptor autoantibodies have occasionally been detected in patients with seemingly unrelated neurological syndromes such as forms of limbic encephalitis, epilepsy and neuromyelitis optica, as well as rare healthy individuals (Brenner *et al.*, 2013; Woodhall *et al.*, 2013; Carvajal-Gonzalez *et al.*, 2014; Ekizoglu *et al.*, 2014; Zuliani *et al.*, 2014; Martinez-Hernandez *et al.*, 2015; Crisp *et al.*, 2017; Lang and Prüss, 2017).

To investigate for the first time, the effects of glycine receptor autoantibodies on glycinergic neurotransmission, we used whole-cell patch clamp electrophysiology in cultured spinal motor neurons. Miniature synaptic currents in cultured motor neurons were rapidly and profoundly decreased following incubation in purified IgG from patients, but not from control subjects, providing compelling evidence for a pathogenic role in PERM. We further determined that autoantibodies from at least some patients exert a direct effect on glycine receptor function.

## Materials and methods

### Patients and samples

Four glycine receptor antibody-positive patients (Patients 1–4) were identified with PERM or stiff person spectrum disorder (clinical details in Table 1). All four had raised serum titres of glycine receptor antibodies (Table 1), as determined by a live cell-based assay (Oxford University Neuroimmunology Service). IgG was purified from serum (Patient 1) or plasma (Patients 2–4). Glycine receptor antibodies were also present in the CSF in Patient 2 (not tested in the other patients). IgG was also purified from serum from four healthy control subjects (Controls 1–4) without detectable glycine receptor antibodies in the live cell-based assay. None of the control or patient IgG samples had detectable AMPA receptor (GluR1 or GluR2) or NMDA receptor antibodies on live cell-based assays (Oxford University Neuroimmunology Service). The samples used in this study were covered by ethical approvals RECA 07/Q1604/28 and 16/YH/0013. The clinical details and serial antibody levels of Patient 2 were reported previously (Stern *et al.*, 2014).

**Table 1:** Clinical features of patients from whom IgG was purified

	Patient 1	Patient2	Patient 3	Patient 4
Age at presentation	61	40	29	60
Gender	Female	Male	Female	Male
Muscle stiffness	Limb rigidity	Limb and axial rigidity	Limb and axial spasms and cramps	Limb and axial rigidity and spasms
Myoclonus/startle	Yes	Yes	No	Yes
Brainstem signs	Swallowing dysfunction, respiratory failure	Swallowing impairment, ophthalmoplegia, respiratory failure	No	Diplopia (no overt ophthalmoplegia), unilateral reduction in hearing and palatal elevation, apnoeic episodes
Cognitive symptoms	Encephalopathy	Preceding mood change and hallucinations but no overt cognitive disturbance	Anxiety and emotional lability but no overt cognitive disturbance	Encephalopathy
Autonomic features	None	Sweating, tachycardia	Urinary retention, constipation, sweating	Urinary retention, constipation, blood pressure lability
Other features	–	–	–	Gait ataxia, dysarthria, pruritus
History of autoimmunity	Type 1 diabetes	None	None	None
History of malignancy	Breast	None	None	None
Estimated titre of purified IgG samples	1:1250	1:500	1:150	1:150

Samples were initially centrifuged and filtered to remove particulate material. IgG was then extracted on Sepharose® G beads according to the manufacturer’s protocol (Protein G GraviTrap™, GE Healthcare), concentrated by ultracentrifugation (Pierce Protein Concentrator PES, 10K MWCO ThermoFisher #88527) and dialysed (Slide-A-Lyzer G2 Dialysis Cassettes, 10K MWCO ThermoFisher #87731) against phosphate-buffered saline (300× volume over 48 h). The final concentration of IgG was measured using a NanoDrop™ and checked by radial immunodiffusion (IgG NL RID, Binding Site #RN004.3). IgG samples were stored at concentrations of 4–10 mg/ml, pH 7.4, −80°C in small volume aliquots to avoid repeat freeze-thaw cycles. Purified IgG was used at a final concentration of 0.5 mg/ml (equivalent to ∼1:30 dilution of serum) for electrophysiological studies, and 0.5 mg/ml or 0.075 mg/ml (equivalent to ∼1:200 dilution of serum) for immunofluorescence.

To generate purified IgG depleted of glycine receptor autoantibodies, 1 mg of purified IgG from Patient 4 was diluted in 1 ml of motor neuron growth media and passaged successively through 30 wells of confluent HEK293 cells expressing alpha1 glycine receptors (passaging was performed in a 24-well plate, 30 min per well). As a control (i.e. no specific depletion of glycine receptor autoantibodies) a 1 mg/ml sample of IgG from Patient 4 was passaged through 30 wells of HEK293 cells expressing LGI1. The resulting depleted IgGs were used at a final estimated concentration of 0.25 mg/ml for immunofluorescence.

Fab fragments were generated from purified IgG samples using a Pierce kit (#44985) according to the manufacturer’s protocol. Samples of the original IgG, products of digestion and purified Fab fragments were separated on a Bis-Tris 4–12% acrylamide gel in MOPS buffer and semidry transferred on a 0.2 µm nitrocellulose membrane, and then labelled with Ponceau S to check for complete digestion and clean Fab purification. The protein ladder used to compare the size was Novex Sharp pre-stained protein standard. Fab fragments were used at a final concentration of 0.2 mg/ml (similar molarity to whole IgG preparations used in the electrophysiology experiments).

### Dissociated spinal cord cultures

Pregnant Sprague Dawley rats were euthanized with CO_2_ to obtain embryonic Day 15 embryos. Spinal cords were dissected into cold Hibernate-E media (Gibco A1247601) and the meninges, dorsal root ganglia and dorsal columns carefully removed. The tissue was digested using Neuronal Isolation Enzyme (Pierce 88285) for 9 min at 37°C. The digested tissue was dissociated by trituration in Hibernate-E with 10% foetal bovine serum, 0.4% bovine serum albumin (Sigma A3311) and 0.1 mg/ml DNase (Sigma DN-25). The supernatant was collected and centrifuged at 370*g* for 5 min. The pellet was resuspended in motor neuron growth medium (as described in Graber and Harris, 2013). Neurons were plated on poly-d-lysine (0.05 mg/ml) coated coverslips on a spinal glial cell layer in a 24-well plate at a density of 10 000 neurons per well. One-third of the medium was exchange three times per week. Cytosine arabinoside (1 μM) was added on Day 5 to inhibit glial growth. All experiments were performed at 17–24 days *in vitro* (DIV).

### Immunofluorescence

To check for surface binding of purified IgG, live dissociated spinal cord cultures were incubated in human IgG 0.5 mg/ml for 15 min at room temperature. Cultures were then fixed in 4% paraformaldehyde before secondary labelling with goat anti-human IgG-Alexa 594. Next, cultures were permeabilized with 0.1% Triton™ X before labelling non-phosphorylated neurofilament (which is abundant in alpha motor neurons; Tsang *et al.*, 2000) with mouse anti-SMI-32 (559844, Merck) 1:500 followed by goat anti-mouse IgG-Alexa 405. Imaging was performed on a Zeiss 880 MP confocal microscope with a 40× objective. Laser intensity and gain settings were maintained across all experiments and analysis performed in ImageJ, with identical linear adjustment of contrast and brightness performed across all experiments.

To examine the relationship of surface bound IgG to glycine receptors and AMPA receptors, live dissociated spinal cord cultures were incubated in human IgG purified from patients 0.075 mg/ml for 1 h at room temperature in motor neuron growth media buffered with HEPES. Cultures were then fixed in 4% paraformaldehyde before labelling with goat anti-human IgG-DyLight™ 488 to detect the bound human IgG. Next, cultures were permeabilized with 0.1% Triton™ X before labelling non-phosphorylated neurofilament with mouse anti-SMI-32 (Merck, 1:500) and glycine receptors containing alpha1 subunits with rabbit anti-glycine receptor alpha1 (AB15012, Merck) 1:500 or AMPA receptors with anti-GluR1 (04-855, Merck) 1:1000. Goat anti-mouse IgG-Alexa 405 and goat anti-rabbit IgG-Alexa 647 secondaries were used to visualize SMI-32 and glycine or AMPA receptors respectively. Imaging was performed on a Nikon Eclipse Ti2 confocal microscope with a 100× objective. Laser intensity and gain settings were optimized for each condition. For imaging of individual processes, sampling was performed at approximately double the Nyquist limit in *xy* and *z*, and blind 3D deconvolution performed using NIS-Elements software. Images were subsequently analysed in ImageJ.

### Electrophysiology

Incubations in IgG were performed by the addition of 0.2 mg IgG to a well (final concentration 0.5 mg/ml) for 15 min at room temperature or 2 h, 4 h or 16 h at 37°C. Coverslips were then removed from the culture plate for analysis by patch clamp electrophysiology in standard recording solutions (not containing IgGs). Neurons on the recording set up were visualized using an Evolve Delta EMCCD camera (Photometrics) and Micro-Manager software, connected to an Olympus IX73 microscope with difference interference contrast optics. Motor neurons were identified by their morphology (large soma >25 µM) for recording. Whole-cell patch clamp recordings were made at 29°C with borosilicate glass pipettes prepared on a micropipette puller (Sutter Instruments), which had a resistance of 3–4 MΩ. The extracellular solution contained 125 mM NaCl, 2 mM CaCl_2_, 1 mM MgCl_2_, 2.5 mM KCl, 10 mM HEPES, and 30 mM glucose (pH adjusted to 7.3 with NaOH, 290 mOsm). The intracellular solution contained 130 mM KCl, 10 mM HEPES, 0.2 mM EGTA, 2 mM MgATP, 0.3 mM NaGTP, and 20 mM sodium creatine phosphate (pH adjusted to 7.3 with KOH, osmolarity adjusted to 290 mOsm with sucrose). Neurons were held at −70 mV with a MultiClamp 700B amplifier (Molecular Devices). Membrane currents were filtered at 2 kHz and acquired at 20 kHz with WinEDR (Strathclyde) software. For baseline recordings of spontaneous ‘miniature’ synaptic currents mediated by AMPA- and glycine-receptors, 500 nM tetrodotoxin (TTX), 50 µM d-2-amino-5-phosphonopentanoate (AP5) and 10 µM bicuculline were included in the extracellular solution. After recording for 3 min, 10 µM 2,3-dihydroxy-6-nitro-7-sulfamoyl-benzo[*f*]quinoxaline-7-sulfonamide (NBQX) was added to the extracellular solution to block AMPA receptors and isolate the glycine receptors. After allowing time for wash-in, a further 3-min recording of miniature synaptic currents mediated by glycine receptors was obtained. Cells with a resting membrane potential more depolarized than −50 mV or showing >30% change in series resistance during the course of recording were rejected. Recordings were made from a minimum of five cells for each experimental condition (time point and IgG sample) obtained from at least two independent cultures, except where indicated (for some Fab experiments that were limited by the availability of sample).

Miniature synaptic currents were analysed using the MiniAnalysis Program (Synaptosoft), with an amplitude threshold 40 pA. All events were visually inspected to verify that they had a rapid rise and slow decay typical of synaptic currents. For each cell the first 500 events or, where there were <500 events all events occurring over 3 min of recording, were analysed. Cells were excluded from further analysis if the miniature events occurred at a frequency of <0.5 Hz or >20 Hz prior to the addition of NBQX.

Charge transfer per unit time through glycine receptors was calculated from the product of the mean area of spontaneous miniature events and event frequency recorded after the addition of NBQX to the bathing solution. The AMPA receptor-mediated charge transfer per unit time was estimated by subtracting the glycinergic charge transfer from the pre-NBQX baseline. For investigations of decay time constant, overlapping events were first removed by visual inspection, then the amplitude-scaled miniature events were averaged for each cell before and after the addition of NBQX. The 10–90% decay phases of these scaled average miniature events were fitted with a double exponential function. The weighted decay time constant was then calculated using the formula:

(1) $$\frac{A_{1}\tau_{1\;}\;+\;A_{2}\tau_{2}}{A_{1}\;+\;A_{2}}$$

### Statistical analyses

Electrophysiological measurements were analysed with Kruskal-Wallis followed by Dunn’s *post hoc* tests, or Mann Whitney U-tests as appropriate. The alpha level used to determine significance was *P* < 0.05. All tests were performed in OriginPro 2018b. Mean values are presented with standard error of the mean (SEM).

### Data availability

All data that support the findings of this study are available upon request from the corresponding author. 

## Results

### IgG from patients profoundly disrupts glycinergic currents

**Figure 1:**
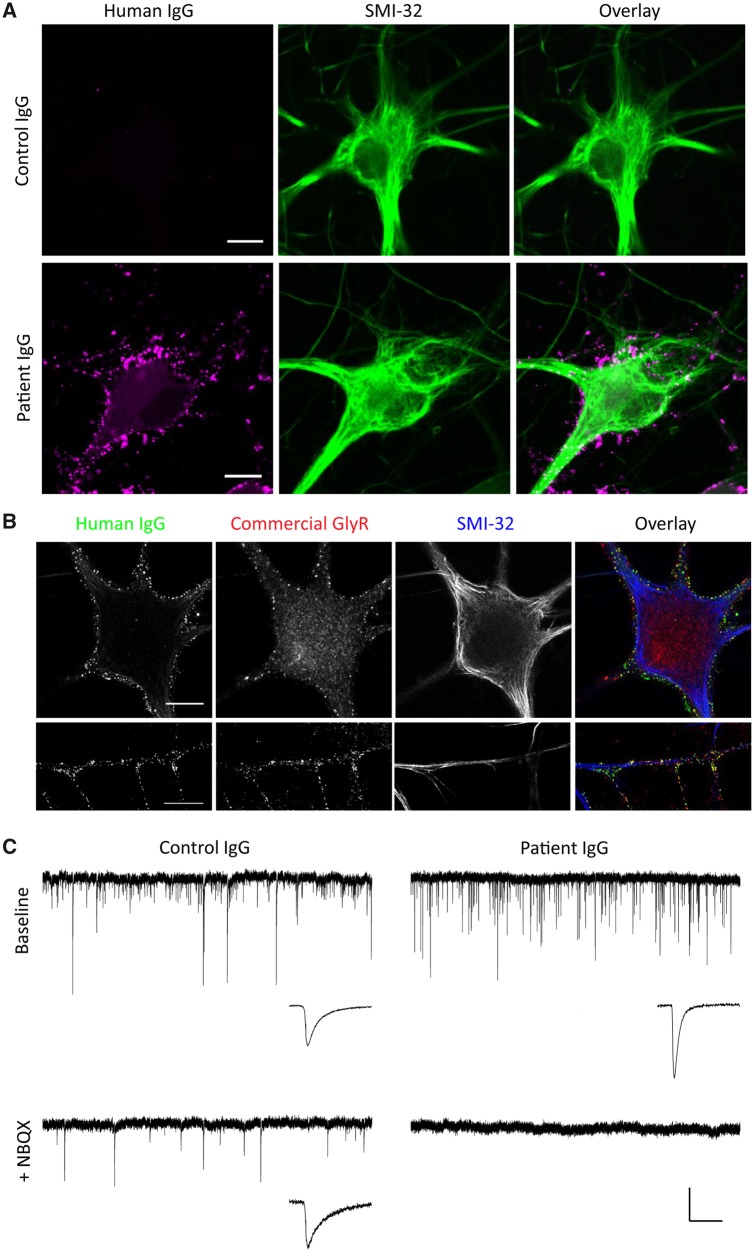
**Whole cell patch clamp recordings of glycinergic mIPSCs.** (**A**) Representative images of purified IgG from patient (Patient 1) or control subjects binding to the surface of cultured motor neurons. Scale bar = 10 µm. (**B**) Representative images of purified IgG from patient (Patient 1) binding to the surface of cultured motor neurons (green) co-stained for alpha1 subunits of glycine receptors with a commercial antibody (red). *Lower panels* show *z*-projections of individual processes. Scale bar = 10 µm. (**C**) Representative traces of miniature postsynaptic currents from whole cell patch clamp recordings of motor neurons following treatment with 0.5 mg/ml IgG purified from patient or control subjects for 2 h. Baseline recordings were made in the presence of TTX, AP5 and bicuculline (*upper traces*). NBQX was added to block AMPAR-mediated glutamatergic currents and thereby reveal the glycinergic mIPSCs (*lower traces*). *Insets* show the average miniature currents recorded in each condition. Vertical scale bar = 50 pA, horizontal scale bar = 1 s (traces) or 10 ms (insets).

We first asked if IgG purified from patients with glycine receptor autoantibodies binds to murine glycine receptors. We incubated live 17–24 DIV dissociated rat spinal cord cultures in IgG 0.5 mg/ml for 15 min at room temperature, and then fixed briefly before looking for IgG binding by immunofluorescence on the fixed but unpermeabilized cells. The cells were subsequently permeabilized and labelled with SMI-32, an antibody to a non-phosphorylated neurofilament, to identify motor neurons. All four IgG samples purified from patients showed strong binding, whereas specific binding of IgG to motor neurons was not observed for any of the control IgG samples (for example, see Patient 1: Fig 1A and Supplementary Fig. 1).

We verified that the patient samples bound to glycine receptors on motor neurons by first incubating live neurons in patient IgGs then fixing and permeabilizing cells to allow co-labelling with a commercial antibody for glycine receptors. The commercial antibody targets the intracellular domain of glycine receptors and therefore labels both surface and internalized receptors. All four patient IgGs showed substantial overlap with the commercial glycine receptor antibody on the surface, but not intracellularly, consistent with the samples containing antibodies that bind to glycine receptors (Fig. 1B and Supplementary Fig. 2).

To assess whether patient IgG affects glycinergic currents we made whole cell patch clamp recordings of miniature synaptic currents from motor neurons incubated in patient or control IgG (Fig. 1B). To verify that recorded motor neurons were synaptically innervated we initially recorded mixed AMPA receptor-mediated miniature excitatory postsynaptic currents (AMPAR mEPSCs) and glycine receptor-mediated miniature inhibitory postsynaptic currents (mIPSCs) in the presence of TTX (a sodium channel blocker used to abolish action potentials), AP5 (an NMDAR receptor antagonist) and bicuculline (a GABA_A_ receptor antagonist). Because the pipette solution contained a high chloride concentration both AMPAR-mediated mEPSCs and glycine receptor-mediated mIPSCs were inward at the holding potential (−70 mV). These currents were not obviously different between control and patient IgG treated cells (Fig. 1B). After the initial recording of 3 min, NBQX was then added to the perfusion solution to isolate pharmacologically the glycinergic mIPSCs (Fig. 1B). After NBQX was added, all remaining miniature currents were abolished by the addition of 400 nM strychnine, indicating that they were completely dependent on glycine receptors (not shown). In the two neurons illustrated (Fig. 1B), the results for incubation in patient IgG, but not in control IgG, were similar to those in strychnine; all glycinergic synaptic activity was abolished after 2-h incubation at 37°C. 

**Figure 2:**
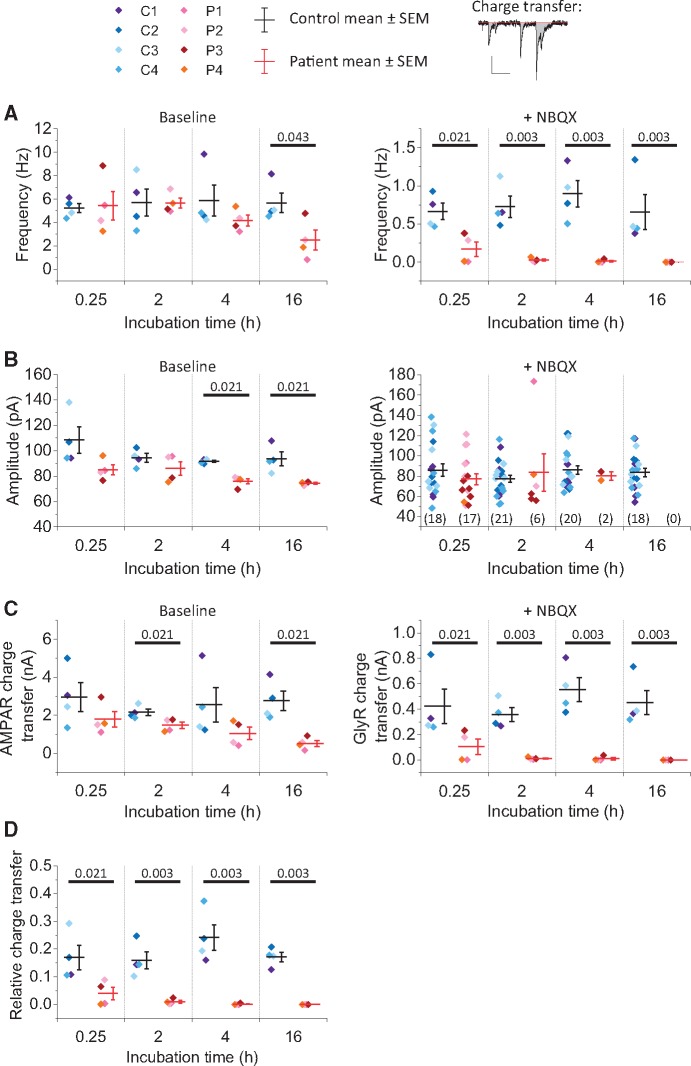
**IgGs from patients with glycine receptor autoantibodies disrupt glycinergic neurotransmission.** (**A**) Box plots show combined mEPSC/mIPSC frequency before NBQX (*left*) and glycinergic mIPSC frequency after the addition of NBQX (*right*) at each time point (mean ± SEM). Each data point represents a single IgG sample (control samples C1–4 and patient samples P1–4). (**B**) *Left* box plot shows mean combined mEPSC/mIPSC amplitude before NBQX for patient and control IgG samples. *Right*: mIPSC amplitude after NBQX is shown for cells where two or more mIPSCs were recorded. (**C**) Box plots show calculated AMPAR-mediated charge transfer per unit time (calculated by subtracting the glycinergic charge transfer from the total charge transfer before the addition of NBQX, *left*) and GlyR charge transfer (*right*). Charge transfer was calculated from the area of all miniature events divided by time (*upper panel*, vertical scale bar = 50 pA, horizontal scale bar = 100 ms). (**D**) Box plot showing mean GlyR:AMPAR mediated charge transfer (individual data points = IgG samples, horizontal bar = mean, whiskers = SEM). (**A**–**D**) Each data point shows the IgG sample mean (calculated from the mean of at least five cells measured following incubation in that particular IgG), horizontal bar indicates the mean of the IgG samples and whiskers indicate SEM (except for mIPSC amplitude after NBQX where *n* = number of cells where two of more mIPSCs were recorded and is indicated in parentheses; no cells met this criterion for 16 h incubation in patient IgGs). Horizontal bars indicate statistical significance *P* < 0.05 by Kruskal-Wallis with Dunn’s *post hoc* comparison (actual *P*-values shown).

Glycine receptor autoantibodies are predominantly of immunoglobulin G subclasses IgG1 and IgG3 (Carvajal-Gonzalez *et al.*, 2014). These are divalent molecules that are capable of cross-linking and internalizing receptors, which could in turn account for a reduction in glycinergic currents. Since the process of cross-linking and internalization is time- and temperature-dependent we used variable incubation times to investigate the pathogenic mechanisms of patient autoantibodies. Cultures were incubated in IgG for 15 min at room temperature, or for 2 h, 4 h or 16 h at 37°C. Mixed mEPSCs and mIPSCs recorded prior to the addition of NBQX showed a decrease in event frequency when exposed to patient IgGs for 16 h (55.8 ± 14.6% of control, *P =* 0.043), and a decrease in event amplitude when exposed to patient IgG for 4 h (17.1 ± 2.3%, *P =* 0.021) or 16 h (20.6 ± 0.7%, *P =* 0.021), as compared to control IgG (Fig. 2A, for each condition *n* = 4 IgGs, with the mean value calculated for each IgG from analysis of recordings from at least five cells in at least two independent cultures). When restricting attention to pharmacologically isolated glycinergic mIPSCs, incubation in patient IgG samples profoundly reduced the frequency of events at all durations [74.3 ± 14.3% for 15-min incubation (*P =* 0.021), 96.0 ± 1.8% for 2-h incubation (*P =* 0.003), 98.7 ± 1.1% for 4-h incubation (*P =* 0.003), 100.0 ± 0.0% for 16-h incubation (*P =* 0.003)] (Fig. 2A). For incubations of over 2 h, glycinergic mIPSCs were very rare indeed, with two or more mIPSCs detected during the 3-min recording period in only 2 of 45 neurons incubated in patient IgG, but in all 41 neurons incubated in control IgG (*P* < 0.00001, Fisher’s).

As so few cells had any glycinergic mIPSCs after prolonged incubation in IgG purified from patients, we measured peak amplitude in the subgroup of cells that had at least two mIPSCs during the 3-min recording after the addition of NBQX (Fig. 2B). Because the number of cells in this subgroup analysis was small, the analysis was performed for cells rather than IgG samples and the results should be interpreted with caution. While the data suggest that patient IgG has a much more profound effect on glycinergic mIPSC frequency than on amplitude, it is not possible to exclude the possibility that incubation in patient IgGs results in a larger proportion of small mIPSCs that fall below the threshold for detection (40 pA), thereby accounting for the apparent reduction in mIPSC frequency observed.

The average charge conducted through glycine receptors was calculated from the total area of all events divided by the duration of recording after the addition of NBQX (Fig. 2C). The average charge conducted through AMPA receptors was calculated by subtracting the average charge conducted through glycine receptors from the average charge conducted before the addition of NBQX (events therefore corresponding to activation of both AMPA receptors and glycine receptors). There was a profound reduction in the charge transfer per unit time through glycine receptors at all durations of incubation [75.3 ± 14.1% for 15-min incubation (*P =* 0.021), 96.7 ± 1.3% for 2-h incubation (*P =* 0.003), 98.2 ± 1.6% for 4-h incubation (*P =* 0.003), 100.0 ± 0.0% for 16-h incubation *P =* 0.003] and a significant reduction in the charge conducted through AMPARs for 2-h (31.7 ± 7.6%, *P =* 0.021) and 16-h (80.9 ± 5.7%, *P =* 0.021) incubation.

Given that we observed effects on AMPAR-mediated miniature currents in addition to the effects on glycinergic events, we looked for binding of patient IgGs to GluR1 or GluR2 AMPAR subunits using a live cell-based assay. None of the IgG samples contained detectable autoantibodies to the AMPAR subunits. Additionally, when live motor neurons were incubated in the purified patient IgG samples, the pattern of staining did not overlap substantially with that for a commercial antibody to the GluR1 subunit of AMPARs (Supplementary Fig. 3), in contrast to the overlap seen with a commercial antibody to the alpha1 subunit of glycine receptors (Fig. 1B and Supplementary Fig. 2). For one of the samples (IgG from Patient 4, chosen because this was the IgG with the largest effect on AMPAR charge transfer) we depleted the sample of autoantibodies to glycine receptors by passaging over HEK293 cells expressing alpha1-glycine receptors. This depleted IgG showed minimal residual binding to motor neurons, and the remaining binding clearly co-localized with glycine receptors identified using a commercial antibody (Supplementary Fig. 4). By contrast, when a second aliquot of the same sample was passaged over HEK293 cells expressing LGI1, the resulting IgG showed substantial binding to motor neurons, co-localizing with glycine receptors. Collectively these experiments rule out the co-existence of other autoantibodies in the purified IgG samples that could explain the effects observed on AMPARs, and argue instead for indirect effects on AMPAR currents (see ‘Discussion’ section).

To adjust the data to account for any potential differences in cell size or overall synapse number, the glycinergic charge transfer per unit time was expressed as a proportion of the calculated AMPAR-mediated charge transfer per unit time (Fig. 2D). The relative glycinergic charge transfer was decreased at all time points [for 15-min incubation 76.3 ± 13.1% reduction compared to control (*P =* 0.021), 2-h incubation 93.5 ± 3.1% (*P =* 0.003), 4-h incubation 99.4 ± 0.5% (*P =* 0.003), 16-h incubation 100.0 ± 0.0% (*P =* 0.003)]. While glycinergic transmission was profoundly diminished for all samples after 2-h incubation in patient IgG, the effect after 15-min incubation was much more marked for IgG from Patients 1 and 4 (99.0 ± 1.0% and 99.6 ± 0.4% reduction compared to control, respectively) than Patients 2 and 3 (78.2 ± 7.4% and 83.9 ± 9.4% of control, respectively).

**Figure 3:**
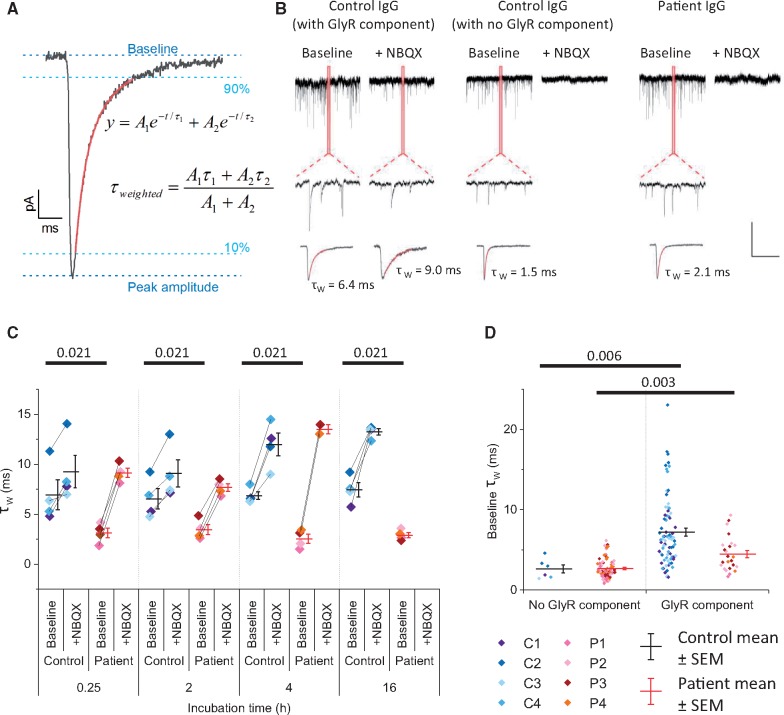
**IgGs from patients with glycine receptor autoantibodies alter the shape of average mIPSCs/mEPSCs.** (**A**) Calculation of weighted tau from the fit of the 10–90% of the decay phase with a double exponential function (shown in red). (**B**) *Top row* shows example traces from three cells recorded before the addition of NBQX (*left panel* for each cell) or after NBQX (*right panel* for each cell) and expanded inset to show difference in individual mIPSC/mEPSC time course. *Bottom row* shows amplitude scaled average miniature events, with the 10-90% decay phase fitted with a double exponential function (red) to calculate the weighted tau (shown). Scale bar = 50 pA, 1 s (*top panel*), 50 ms (*middle panel*) and 10 ms (*bottom panel*). (**C**) Weighted tau for patient and control IgGs before and after addition of NBQX. Bars = mean values, error bars = SEM. Horizontal bars indicate statistical significance *P* < 0.05 by Kruskal-Wallis with Dunn’s *post hoc* comparison. (**D**) Weighted tau for cells separated by the presence or absence of glycinergic mIPSCs after the addition of NBQX. Horizontal bars indicate statistical significance *P* < 0.05 by Kruskal-Wallis with Dunn’s *post hoc* comparison.

Because glycinergic currents decay more slowly than AMPAR-mediated currents, we examined the effect of IgG on the average shape of mixed mEPSCs/mIPSCs prior to the addition of NBQX, and of isolated glycinergic mIPSCs (Fig. 3). As expected, the addition of NBQX led to an increase in weighted tau. Prior to the addition of NBQX, neurons incubated in control IgG had longer decay time constants than neurons incubated in patient IgG at all time points [3.1 ± 0.5 ms compared with 6.9 ± 1.5 ms for 15-min incubation (*P =* 0.021), 3.5 ± 0.5 ms compared with 6.5 ± 1.0 ms for 2-h incubation (*P =* 0.043), 2.5 ± 0.5 ms compared with 6.9 ± 0.4 ms for 4-h incubation (*P =* 0.021), 2.9 ± 0.3 ms compared with 7.4 ± 1.4 ms for 16-h incubation (*P =* 0.021)] (Fig. 3B and C). By contrast, the average shape of miniature events recorded prior to addition of NBQX was similar between neurons incubated in patient IgG and those rare neurons incubated in control IgG that did not have a glycinergic component (2.7 ± 0.1 ms for patient IgG compared with 2.6 ± 0.5 ms for control IgG, Fig. 3D).

Collectively, these data demonstrate the presence of circulating IgG autoantibodies in all four patient IgG samples that severely disrupt glycinergic neurotransmission and could thereby account for the neurological symptoms observed in these four patients. The observation that glycinergic currents are disrupted by short incubations in patient IgG at room temperature was surprising, and suggested that antibody-mediated cross-linking and internalization of glycine receptors may not be the only or even the main pathogenic mechanism.

### Fab fragments from some patients disrupt glycinergic mIPSCs

**Figure 4:**
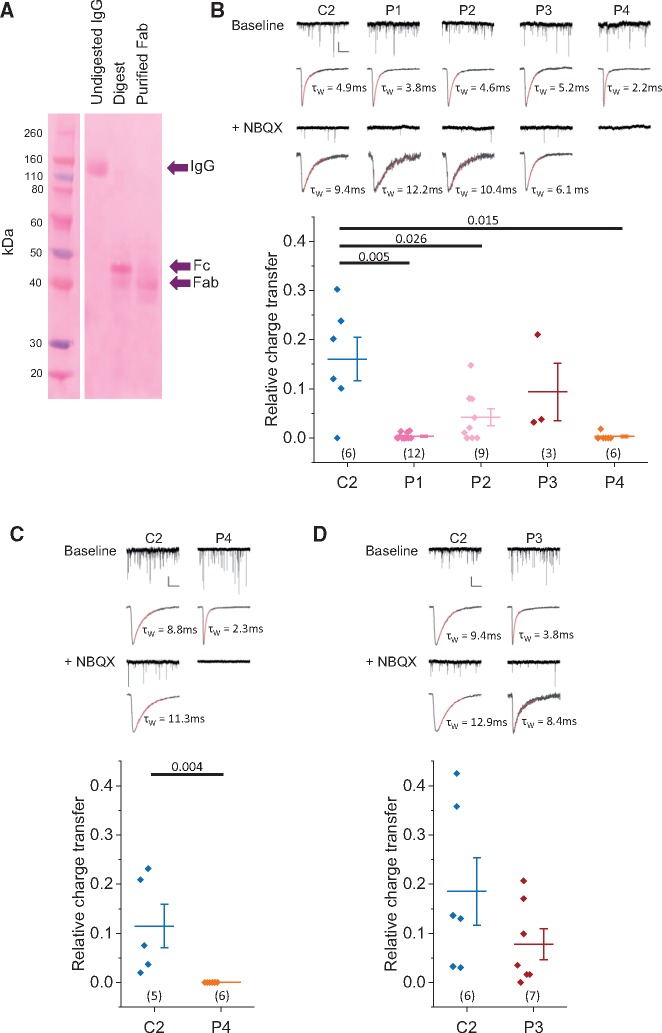
**Fab fragments from some patients disrupt glycinergic neurotransmission.** (**A**) Example electrophoresis gel stained with Ponceau S showing a protein ladder (*left*), the undigested IgG, digest (containing Fc and Fab fragments) and purified Fab fragments. (**B**) *Upper panel* shows example traces and example amplitude scaled average miniature events from cells incubated in Fab fragments generated from IgGs from Control Subject 2 and Patients 1–4 (patients). Box plot shows relative (GlyR:AMPAR) charge transfer. (**C**) Example traces, example scaled average miniature events and box plot of relative charge transfer for neurons previously incubated in Fab fragments from Control Subject 2 or Patient 4 for 15 min. (**D**) Example traces, example scaled average miniature events and box plot of relative charge transfer for neurons incubated in Fab fragments generated from Control Subject 2 or Patient 3 for 16 h. (**B**–**D**) Vertical scale bar = 50 pA, horizontal scale bar = 1 s for example traces and 10 ms for example scaled miniature events. Horizontal bar = mean, whiskers = SEM, numbers in parentheses = number of cells. Horizontal bars indicate statistical significance *P* < 0.05 by two-tailed Mann-Whitney U-tests.

The half-life of surface glycine receptors has been reported as 14–48 h in cultured murine motor neurons (Hoch *et al.*, 1989; Rasmussen *et al.*, 2002). However, glycine receptor internalization may be accelerated by antibody binding, as suggested by observations in HEK293 cells over-expressing glycine receptors in which patient IgG resulted in significant loss of receptors within 2 h (Carvajal-Gonzalez *et al.*, 2014). Downregulation of other cell surface receptors by autoantibodies, for example NMDA receptors in anti-NMDAR encephalitis and acetylcholine receptors in myasthenia gravis, has been shown to depend upon divalency of the IgG1 molecules (Drachman *et al.*, 1978; Hughes *et al.*, 2010). We therefore generated monovalent Fab fragments from patient IgG to investigate whether receptor cross-linking is required for pathogenicity (Fig. 4A).

Dissociated spinal cord cultures were incubated in patient Fab fragments for 2 h. Fab fragments generated from purified IgG from Patients 1, 2 and 4 disrupted glycinergic neurotransmission (98.2 ± 0.9% reduction, Mann-Whitney U = 64, *P =* 0.005; 74.7 ± 10.1%, Mann-Whitney U = 45.5, *P =* 0.026; and 79.1 ± 18.4%, Mann-Whitney U *=* 32.5, *P =* 0.015, respectively) (Fig. 4B). Incubation in Fab fragments from Patient 3 did not abolish glycinergic transmission: although the relative glycinergic charge transfer was smaller than that observed after incubation in one of the control IgG samples, this difference fell short of significance (44.0 ± 14.6%, Mann-Whitney U = 13.5, *P =* 0.286). These data show that the severe reduction in glycinergic currents observed following incubation in IgGs from Patients 1, 2 and 4 does not require divalency of the antibodies, suggesting that the mechanism does not require cross-linking of glycine receptors. A 15-min room temperature incubation using Fab from Patient 4 showed that disruption of glycinergic transmission with Fab fragments from this patient occurs rapidly, as was observed for whole IgG (Fig. 4C), implying a direct blocking effect on glycine receptors.

IgGs from Patients 2 and 3 showed less disruption of glycinergic neurotransmission at 15 min than IgGs from Patients 1 and 4 (Fig. 2D). To examine whether the reduction in glycinergic current with more prolonged incubations observed with IgG from Patient 3 depended upon divalency, we incubated neurons in Fab fragments from Patient 3 for 16 h. The small, non-significant, decrease in relative glycinergic signalling (58.1 ± 16.9%, Mann-Whitney U = 30, *P =* 0.224; Fig. 4D) was no larger than that observed after 15 min (Fig. 4B). We tentatively conclude that the larger effect on glycinergic transmission observed with more prolonged incubation in whole IgG from Patient 3 (100% after 16 h, Fig. 2D) indeed depends on the ability of the antibodies to cross-link receptors, which cannot occur when the Fab fragments bind.

## Discussion

Although PERM and SPS have long been considered disorders of inhibitory synaptic transmission (Meinck and Thompson, 2002) we provide the first direct demonstration that autoantibodies that bind glycine receptors in patients with these disorders disrupt glycinergic currents in spinal motor neurons.

Most autoantibodies to neuronal receptors are thought to act by binding divalently to adjacent receptors leading to internalization and loss of the target; this process is both time- and temperature-dependent (Heinemann *et al.*, 1977; Drachman *et al.*, 1978; Toyka *et al.*, 1980; Hughes *et al.*, 2010). The known exceptions are autoantibodies to GABA_B_ receptors and foetal acetylcholine receptors (Riemersma *et al.*, 1996; Nibber *et al.*, 2017) that inhibit receptor function directly. Previous work has shown that glycine receptor antibodies from patients were internalized when applied to HEK293 cells expressing alpha1 homomeric glycine receptors, consistent with receptor cross-linking and depletion from synapses (Carvajal-Gonzalez *et al.*, 2014). However, our experiments in which the effects of IgG purified from individual patients were studied have shown pathogenic consequences on short time scales and, in the case of three out of four patients, these occur even with Fab fragments that cannot cause cross-linking and internalization. The molecular mechanisms of glycine receptor autoantibodies, therefore, probably include direct antagonistic actions for example by steric hindrance or conformational loss of the ligand binding site or disruption to the chloride channel, or alternatively an effect on glycine receptor within-membrane mobility.

Purified IgGs from patients had a profound effect on mIPSC frequency in the present study. This could have been because the mIPSC amplitudes were reduced resulting in the majority of events falling below the threshold for detection. However, we observed little effect on mIPSC amplitude (though only relatively few events were detected after incubation in patient IgGs) suggesting either that smaller clusters of glycine receptors are more vulnerable to antibody-mediated disruption than large clusters, or that pathogenicity includes presynaptic as well as postsynaptic mechanisms. Mutations in presynaptic glycine transporters (predominantly GlyT2) are the second most common cause of hereditary hyperekplexia (Rees *et al.*, 2006; Carta *et al.*, 2012), but targeted searches for GlyT2 autoantibodies in patients with PERM/SPS have found few cases (Martinez-Hernandez *et al.*, 2016). Interestingly, however, a substantial presynaptic contribution to the phenotype of hereditary hyperekplexia caused by a point mutation in alpha1 glycine receptors has recently been proposed (Xiong *et al.*, 2014). In addition to the dramatic effects we observed on glycinergic currents, we also observed a reduction in AMPAR mediated neurotransmission with prolonged incubation in patient IgGs (Fig. 2C). This raises the possibility that compensatory changes may occur in this culture system in response to reduced inhibitory neurotransmission; for example a homeostatic reduction in AMPAR expression to maintain the excitatory-inhibitory balance (O'Brien *et al.*, 1998; Ibata *et al.*, 2008). However, as stimulation of presynaptic glycine receptors has been proposed to increase neurotransmitter release by presynaptic terminal depolarization at calyceal synapses in the medial nucleus of the trapezoid body (Turecek and Trussell, 2001) and in the dorsal spinal cord (Jeong *et al.*, 2003), an alternative explanation might be that pathogenic autoantibodies act presynaptically to reduce both glycine and glutamate release.

Glycine receptors can exist either as pentameric alpha subunit homomers or alpha-beta subunit heteromers, and their expression in the nervous system is developmentally and spatially regulated (Lynch, 2009). In adults, most glycine receptors are alpha1-beta heteromers expressed predominantly in the spinal cord and brainstem where, together with GABA_A_ receptors, they mediate fast inhibition. In a previous study, autoantibodies to glycine receptors from patients bound to the extracellular domain of the alpha1 subunit, both in alpha1 homomers and alpha1-beta heteromers (Carvajal-Gonzalez *et al.*, 2014). Antibodies from some patients additionally bound other alpha subunits, but no correlation was found between clinical phenotype and subunit specificity. At present the factors that determine the diverse clinical features in patients with these autoantibodies therefore remain unclear. Our results raise the possibility that autoantibodies with different pathogenic mechanisms may be present in individual patients. In turn, this may account for some of the clinical variability observed in patients with glycine receptor autoantibodies. For example Patient 3, compared with Patients 1, 2 and 4, had a milder clinical course with no brainstem signs, and yielded the only IgG sample for which Fab fragments did not significantly reduce the glycinergic currents.

However, we acknowledge a number of limitations of our investigations. The study was limited to four patients with PERM/SPS, which is insufficient to firmly establish whether the pathogenic mechanism of autoantibodies in individual patients correlates with the clinical features. A further possibility is that there are dose dependent effects of the glycine receptor autoantibodies. IgGs purified from Patient 3 were the lowest titre glycine receptor antibodies on the live HEK293 cell-based assay. As our patient group was restricted to those with PERM/SPS, we are also unable to comment on the pathogenicity of glycine receptor autoantibodies found sometimes in patients with other neurological syndromes, such as seizures or neuromyelitis optica. Correlations between clinical characteristics and pathogenic mechanisms or antibody titres could be further explored in larger patient cohorts using assays of glycine receptor function.

The relative contributions of antigen cross-linking and internalization or more direct effects on receptor function to the clinical picture in patients are also unclear. Other mechanisms may also be important *in vivo*. For example, glycine receptor autoantibodies can fix complement *in vitro* (Carvajal-Gonzalez *et al.*, 2014), although it remains unknown whether they do so in the CNS of patients. Such questions can only be answered with detailed studies in patients or in animal models of disease.

Despite these limitations, the dramatic reduction in glycinergic currents following incubation in patient IgG, strongly supports the hypothesis that glycine receptor autoantibodies in patients with PERM/SPS are pathogenic and are responsible for the clinical phenotypes observed. The finding that glycine receptor autoantibodies in patients can have direct and rapid effects on glycine receptors is intriguing and would suggest that, if this antagonistic action is confirmed to be a dominant mechanism in patients, treatment strategies to deplete autoantibodies or block binding to the antigenic target may be more successful than potential therapeutic strategies targeting internalization pathways or complement cascades.

## Supplementary Material

awz297_Supplementary_Data

## Abbreviations

mEPSC =: miniature excitatory postsynaptic current
mIPSC =: miniature inhibitory postsynaptic current
PERM =: progressive encephalomyelitis with rigidity and myoclonus
SPS =: stiff person syndrome
