# Supplementary material for: Glycine receptor autoantibodies disrupt inhibitory neurotransmission
Source: Brain. 2019 Oct 8;142(11):3398–410. doi: 10.1093/brain/awz297 (PMC6821286; doi:10.1093/brain/awz297)
Supplement: awz297_Supplementary_Data [file awz297_supplementary_data.pdf]

Supplementary figure 1: Binding of patient IgGs to cultured motor neurons

Representative images of purified IgG from patients P1-4 binding to the surface of cultured motor neurons. Scale bar = 10 µm.

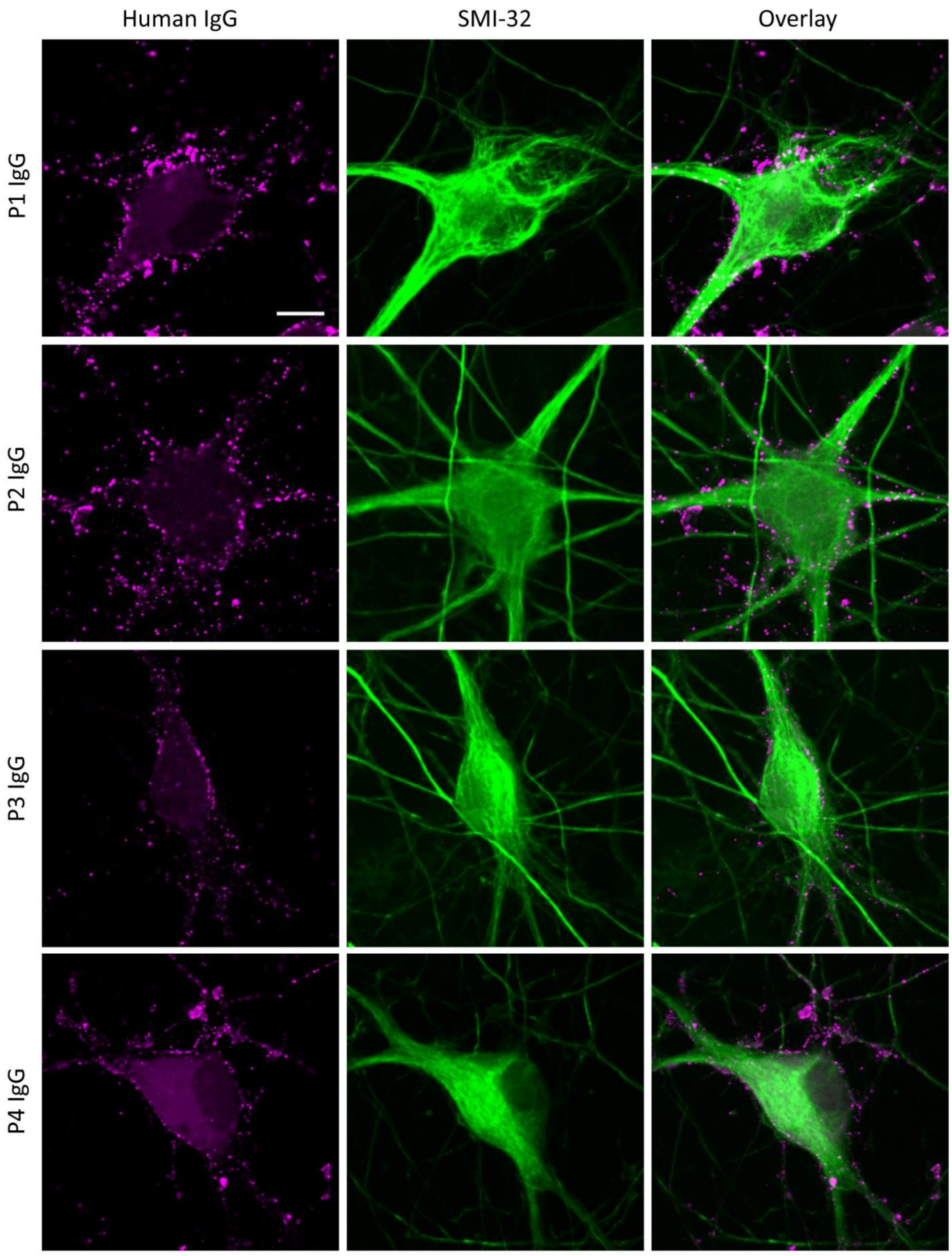
Human IgG
SMI-32
Overlay
P1 IgG
P2 IgG
P3 IgG
P4 IgG

Supplementary figure 2: Patient IgGs bound to the surface of cultured motor neurons co-localise with a commercial glycine receptor antibody

(A) Representative images of purified IgG from patients P1-4 (green) binding to the surface of cultured motor neurons (stained with SMI-32, blue), co-labelled with a commercial alpha-1 glycine receptor antibody (red). (B) Z-maximal projections of individual process labelled with purified IgG from patients P1-4 (green) and a commercial alpha-1 glycine receptor antibody (red). Scale bars = 10 µm.

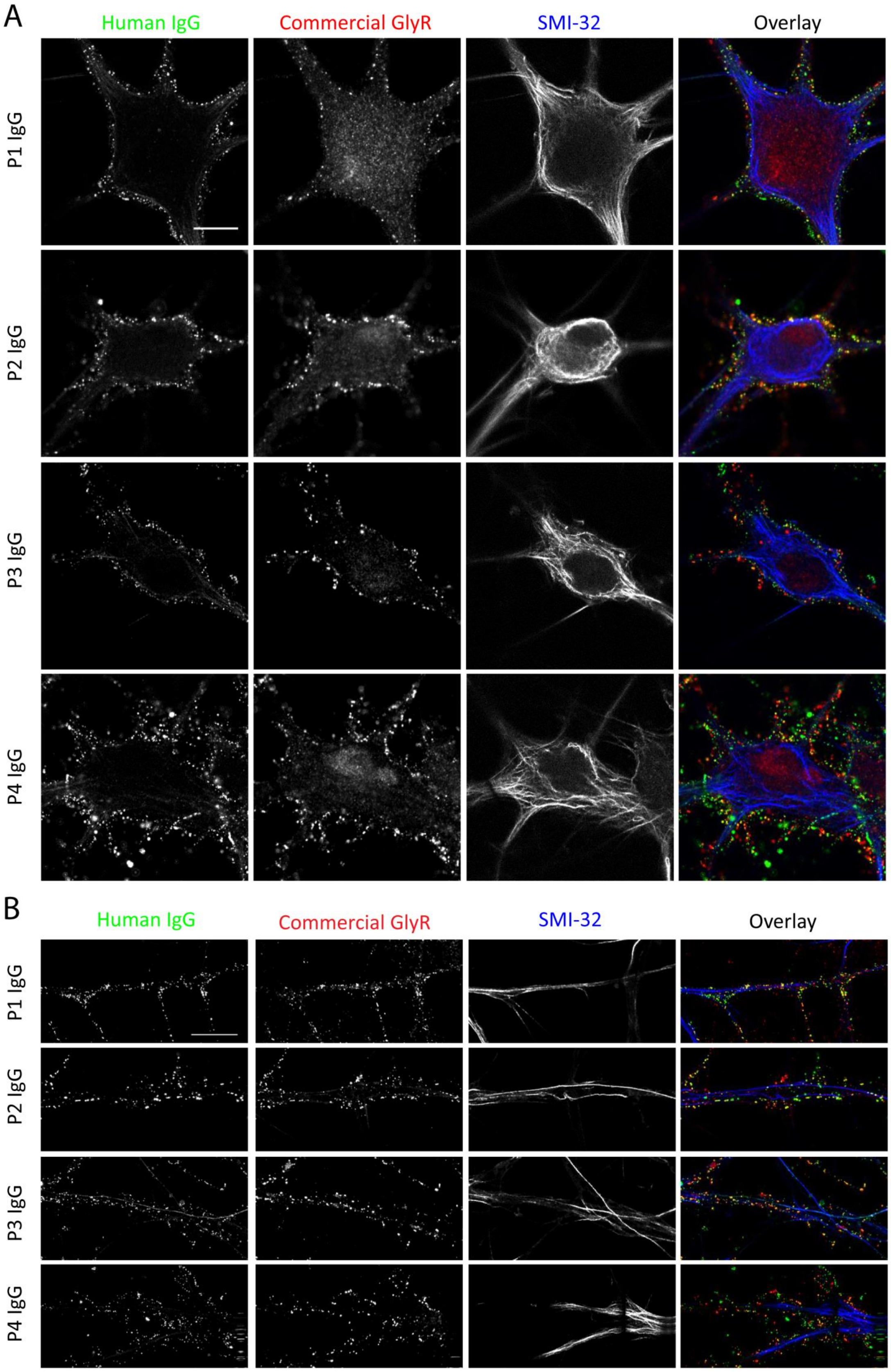
A
Human IgG
Commercial GlyR
SMI-32
Overlay
P1 IgG
P2 IgG
P3 IgG
P4 IgG
B
Human IgG
Commercial GlyR
SMI-32
Overlay
P1 IgG
P2 IgG
P3 IgG
P4 IgG

Supplementary figure 3: Patient IgGs bound to the surface of cultured motor neurons do not co-localise with a commercial GluR1 subunit antibody

(A) Representative images of purified IgG from patients P2-4 (green) binding to the surface of cultured motor neurons (stained with SMI-32, blue), co-labelled with a commercial GluR1 subunit antibody (red). (B) Z-maximal projections of individual processes labelled with purified IgG from patients P2-4 (green) and a commercial GluR1 subunit antibody (red). Scale bars = 10 µm.

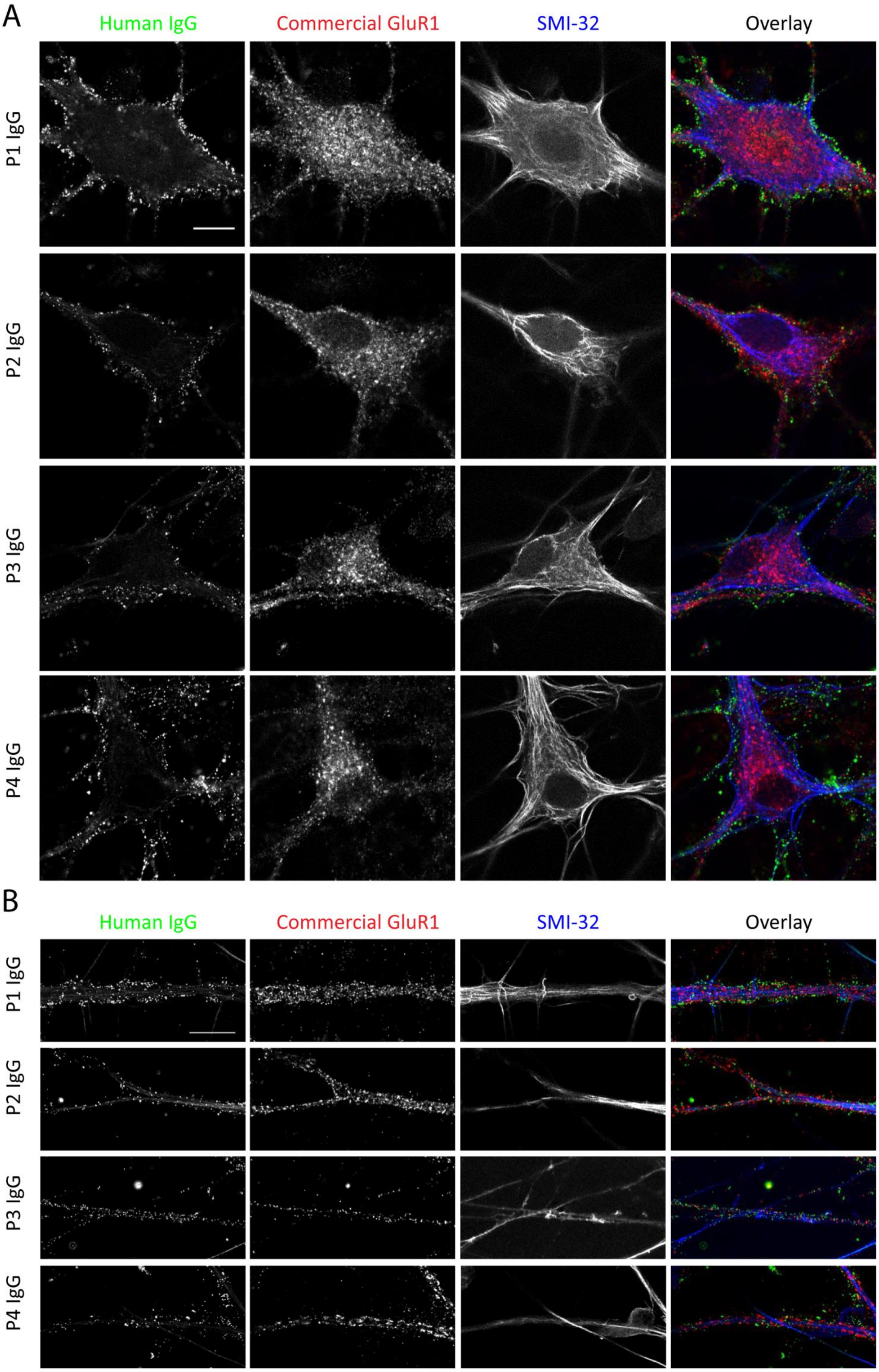
A
Human IgG
Commercial GluR1
SMI-32
Overlay
P1 IgG
P2 IgG
P3 IgG
P4 IgG
B
Human IgG
Commercial GluR1
SMI-32
Overlay
P1 IgG
P2 IgG
P3 IgG
P4 IgG

Supplementary figure 4: Patient IgGs depleted of glycine receptor antibodies do not bind other antigenic targets on motor neurons

(A) Representative images of purified IgG from patients P4 following depletion on HEK293 cells expressing LGI1 (control condition, upper panels) or alpha1 glycine receptors (lower panels). IgG binding is depicted in green, to the surface of cultured motor neurons (stained with SMI-32, blue), co-labelled with a commercial alpha-1 glycine receptor antibody (red). (B) Z-maximal projections of individual processes labelled with purified IgG from P4 depleted on HEK293 cells expressing LGI1 (control) or alpha1 glycine receptors. Scale bars = 10 μm.

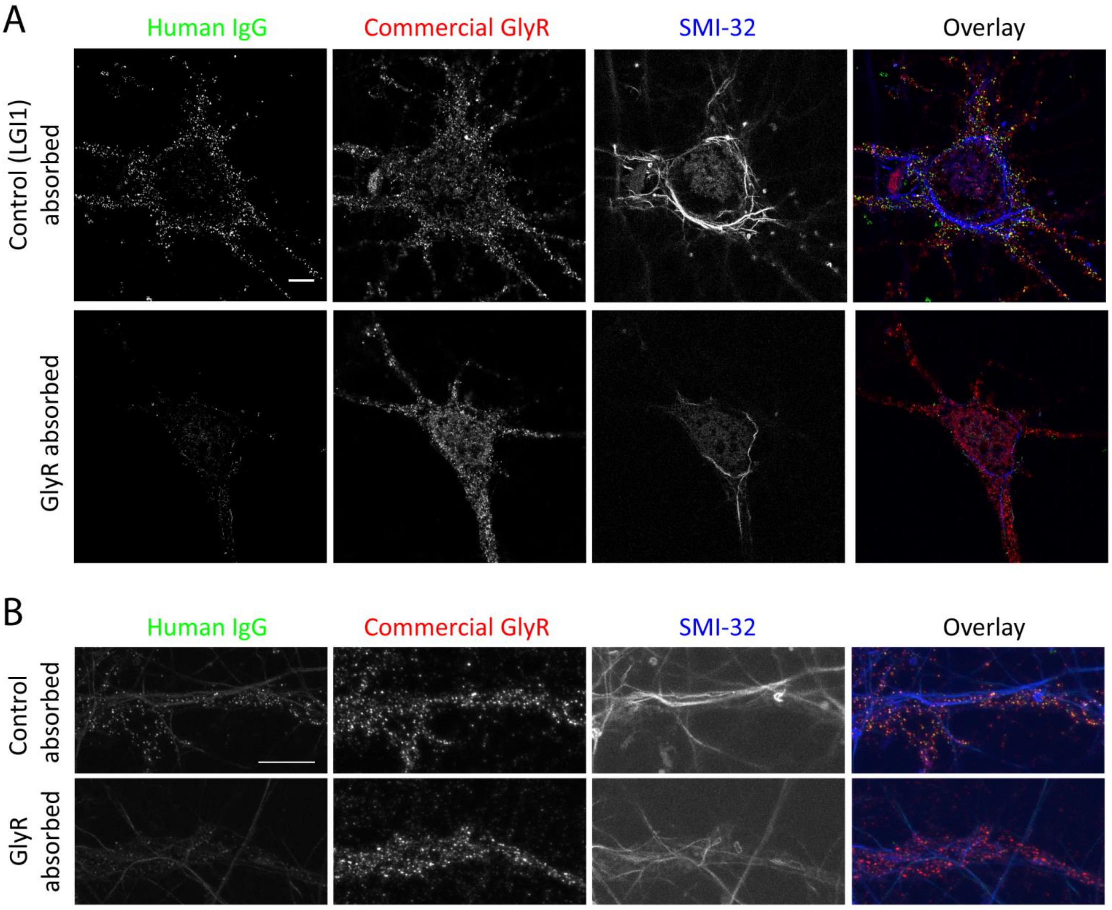
A
Human IgG
Commercial GlyR
SMI-32
Overlay
Control (LGI1) absorbed
GlyR absorbed
B
Human IgG
Commercial GlyR
SMI-32
Overlay
Control absorbed
GlyR absorbed
